# Silencing of the AV2 gene by antisense RNA protects transgenic plants against a bipartite begomovirus

**DOI:** 10.1186/1743-422X-4-10

**Published:** 2007-01-19

**Authors:** Muhammad Mubin, Shahid Mansoor, Mazhar Hussain, Yusuf Zafar

**Affiliations:** 1 Plant Biotechnology Division, National Institute for Biotechnology and Genetic Engineering (NIBGE), P O Box 577, Jhang Road, Faisalabad, Pakistan

## Abstract

Whitefly-transmitted geminiviruses (genus *Begomovirus*) are phytopathogens that cause heavy losses to crops worldwide. Efforts to engineer resistance against these viruses are focused mainly on silencing of complementary-sense virus genes involved in virus replication. Here we have targeted a virion-sense gene (AV2) to develop resistance against *Tomato leaf curl New Delhi virus*, a bipartite begomovirus prevalent throughout the Indian subcontinent. We show that tobacco plants transformed with an antisense construct targeting this gene are resistant to the virus. Following challenged with the virus, transgenic plants remained symptomless, although viral DNA could be detected in some plants by PCR. This is the first report of transgenic resistance against a bipartite begomovirus obtained by targeting a virion-sense gene. The relatively conserved nature of the gene suggests that the technology may be useful to develop broad-spectrum resistance which is required because of the fact that plants are often infected with multiple begomoviruses in the field.

## Findings

### Introduction

The geminiviruses (family *Geminiviridae*) are a group of phytopathogenic viruses having circular, single-stranded (ss) DNA genomes packed within twinned quasi-icosahedral (geminate) particles. These viruses replicate in the nucleus and are transmitted by insect vectors. Vector specificity for transmission is determined by the coat protein [1]. Geminiviruses have been classified into four genera based on insect vectors and genome organization [2]. The whitefly-transmitted geminiviruses are numerous and form the genus *Begomovirus*. These viruses have either two genomic components (DNA A and DNA B) or a single genomic component equivalent to DNA A. Some geminivirus diseases in the Old World are caused by monopartite begomoviruses which require a ssDNA satellite, known as DNA β, to infect and cause symptoms in the hosts from which they were isolated [3,4]. More than 100 species of begomoviruses have been completely sequenced and phylogenetic studies have shown that begomoviruses can be broadly divided into two groups, those originating from the Old World begomoviruses (Europe, Africa, Asia and Australia) and those originating from the New World begomoviruses (the Americas) [5]. However, modern agriculture and trade is disseminating these viruses to new locations. All begomoviruses from the New World are bipartite and lack a virion-sense open reading frame (ORF) designated AV2. This is involved in the movement of monopartite viruses but its function for bipartite begomoviruses is not fully understood [6]. Begomoviruses infecting tomato are the most diverse group and include monopartite, bipartite as well as monopartite begomoviruses that require DNA β.

Begomoviruses are amongst the most devastating phytopathogenic viruses and cause great yield losses throughout the world, particularly in warmer climates that are more suitable for their whitefly vector, *Bemisia tabaci *Genn. Genetically-engineered resistance has the potential to control these viruses and at least some success has been reported [7]. Most of the efforts based on the concept of "pathogen-derived resistance" have been focused on targeting complementary-sense genes involved in virus replication. However, for begomoviruses, these sequences are more diverse than virion-sense genes and thus are less likely to result in a broad-spectrum resistance. Here, we have targeted a virion-sense gene (AV2), using anti-sense RNA technology, and show that transgenic plants are resistant to *Tomato leaf curl New Delhi virus *(ToLCNDV), a bipartite begomovirus that occurs widely across the Indian subcontinent in a number of hosts [8].

### The study

**Figure 1:**
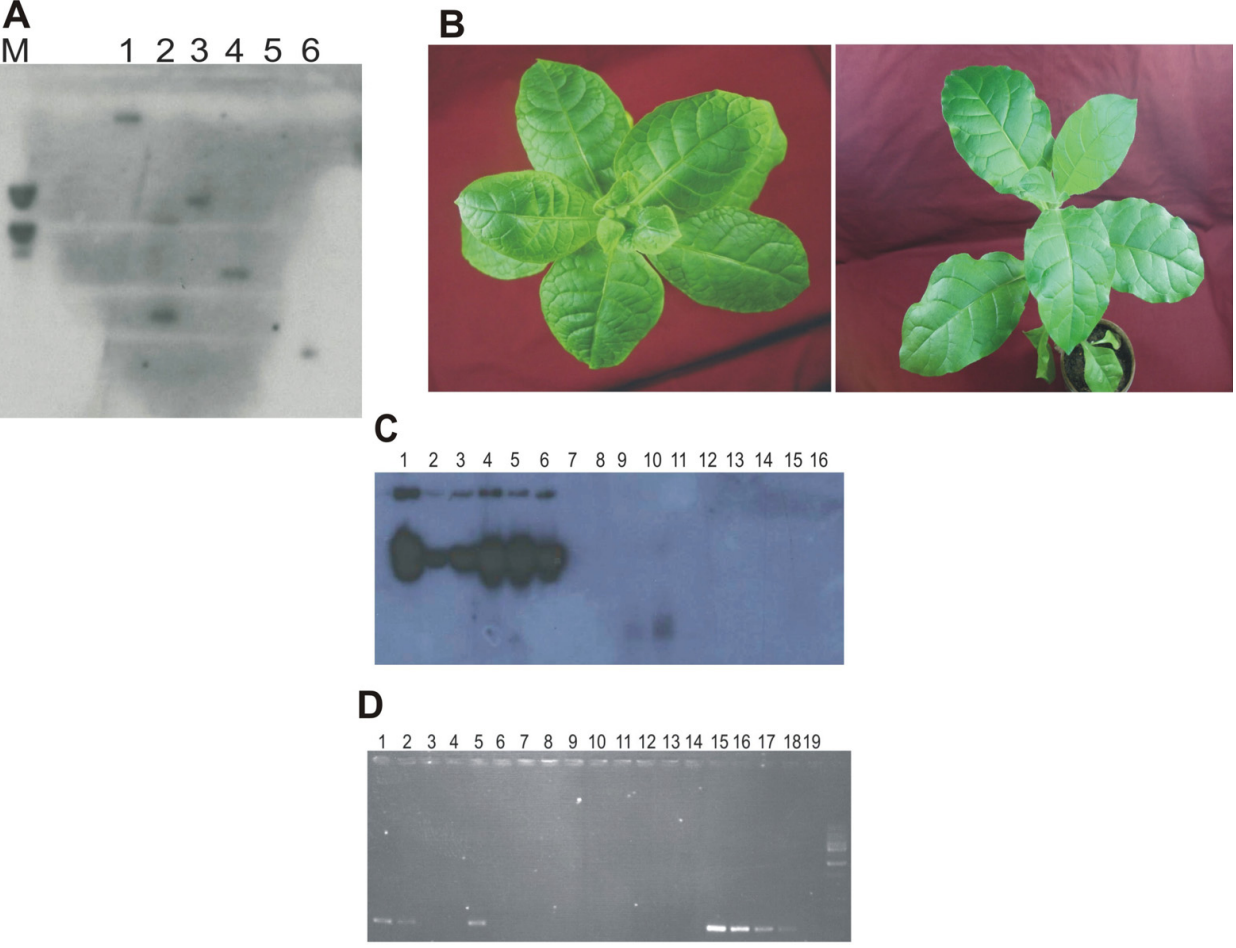
**Transformed *N. tabacum *plants contain the transgene and are resistant to ToLCNDV**. **(Figure1 A) **Southern hybridisation of EcoRI restricted DNA extracted from six primary transformed plants probed for AV2 sequences. Either 1 or 2 hybridising bands were detected in 5 of the 6 samples. **(Figure1 B) **Control *N. tabacum *plants inoculated with cloned ToLCNDV developed symptoms typical of ToLCNDV (left panel). Plants expressing the antisense-AV2 construct remained symptomless (right panel). Photographs were taken 3 weeks post-inoculation. **(Figure1 C) **Southern hybridisation of DNA extracted from control (lanes 1–6) and antisense-AV2 transformed (lanes 7–16) *N. tabacum *plants inoculated with cloned ToLCNDV. The blot was probed for the presence of the DNA A component of the virus. **(Figure1 D) **Serial dilutions of DNA extracted from three antisense-AV2 transformed plants (Lanes 1–12) and controls (Lanes 15–18) were tested for the presence of ToLCNDV by PCR. Dilution is made ten folds. In two transgenic lines the presence of ToLCNDV was detected only at higher template DNA concentrations.

Primers used to amplify the AV2 gene were based on the published sequence of ToLCNDV DNA A (accession number U15015) [8]. The primers ASF (5'-AGGGATCCTGGCTATACATTCTGTACAT-3') and ASR (5'-AGTCTAGAATGTGGGATCCATTATTG-3') having *Bam*HI and *Xba*I restriction endonuclease recognition sequences (underlined) were used in PCR reactions containing the ToLCNDV DNA A clone as the template. The resulting 339 bp PCR product was cloned into a plasmid vector and sequenced. Subsequently, the fragment was directionally cloned at *Bam*HI and *Xba*I sites into the expression vector pFGC5941 [9]. This is a dsRNA (hairpin) vector which in this case was used to express only the AV2 gene in the antisense orientation. The construct was then transformed into *Agrobacterium tumefaciens *strain LBA4404 by electroporation. The transgene and agroinfiltrated gene constructs were sequenced to confirm the integrity of the inserts. *Nicotiana tabacum *(cv. Samsun) was transformed by the *Agrobacterium*-mediated leaf disk method [10]. The putative transgenic plants were selected on ammonium glufosinate (250 mg/l) and assessed for the presence of the transgene by PCR-mediated amplification of the transgene as well as the *Cauliflower mosaic virus *(CaMV) 35S promoter. The number of integration sites of the transgene in plants was determined by genomic Southern hybridization. For this purpose total DNA isolated from six transgenic plants was digested with *Eco*RI (for which there is only a single recognition sequence within the construct) and Southern blotted to nylon membranes. A α^32^PdCTP-labelled probe of ToLCNDV AV2 gene was prepared by the oligo-labelling method and hybridized to the membranes. All six plants tested showed the presence of the transgene with one to two copies (Figure 1A).

Transformed and non-transformed plants were inoculated with the cloned components of ToLCNDV by agroinfiltration, as described previously [11]. Replication of ToLCNDV inoculated plants was assessed by Southern hybridization using the radioactively-labelled full-length clone of ToLCNDV DNA A as a probe. For Southern hybridization total DNA from transgenic lines as well as control plants was isolated and quantified by spectrophotometry. Equal amounts of total DNA was resolved in agarose gels, transferred to nylon membrane and probed with α^32^PdCTP-labelled probe of ToLCNDV DNA A. A semi-quantitative PCR was also done to assess the relative level of viral DNA for plants in which viral DNA could not be detected by Southern hybridization. The primers used, NSPf (5'-GTCCCGGGTATGTCACGTATCCATCA-3') and NSPr (5'-GTATCGATTTATCCAATGTAATTAAGAAT-3'), were designed to amplify the DNA B-encoded nuclear shuttle protein gene and amplify an approximately 550 bp fragment [8].

## Results and conclusion

Ten *N. tabacum *plants, primary transformants resulting from independent transformation events, were selected randomly for further evaluation. All tested plants were positive for the presence of the transgene as well as the CaMV 35S promoter. The presence of the transgene had no apparent adverse effect on the growth of plants, which were morphologically normal and indistinguishable from non-transformed plants. 10 T_0 _transgenic and 5 non-transgenic *N. tabacum *plants were agroinfiltrated with ToLCNDV components and maintained in an insect-proof glasshouse. All 5 non-transgenic plants developed symptoms typical of ToLCNDV, consisting of leaf curling and stunting, within three weeks of inoculation (Fig 1B, left panel). In contrast, no such symptoms were observed on the antisense AV2 transgenic plants (Figure 1B, right panel). Southern blot hybridisation of nucleic acids extracted from systemic (non-inoculated) leaves 25 days after agroinfiltration showed the presence of viral DNA in all 5 non-transgenic plants but not in transgenic plants (Fig 1C).

We further examined the inoculated transgenic plants for the presence of virus by PCR using primers designed to target DNA B (outside the region of the transgene) and serial dilutions of the template DNA (Ten fold dilutions). These analyses detected the presence of virus in four of the ten transgenic plants, but only at low dilutions (high DNA concentrations); whereas in non-transgenic infected plants viral DNA was detected at all dilutions (Figure 1D). These results confirm that antisense AV2 transgenic plants were able to efficiently prevent virus replication and/or movement; with resistance approaching immunity in 6 out of 10 plants.

The aim of this study was to explore the possibility of obtaining resistance to ToLCNDV by silencing the virion-sense AV2 gene. ToLCNDV encodes two genes in the virion-sense, of which only AV2 is essential for the movement of the virus in plants [12]. A recent report has similarly shown that AV2 of *Indian cassava mosaic virus *(ICMV), another bipartite begomovirus from the Indian subcontinent, is involved in virus spread to neighbouring cells [6], whereas the V2 (a homolog of AV2) of a monopartite begomovirus is involved in overcoming host defences mediated by post-transcriptional gene silencing as well as in movement [13,14].

The AV2 sequence is well conserved among begomoviruses from the Old World [12]. This suggests that silencing of AV2 may be a useful strategy for developing broad-spectrum resistance, a highly desirable character in the case of begomoviruses where numerous distinct virus species/strains may cause disease in a particular crop. Our current studies are investigating the spectrum of resistance provided by the antisense construct of ToLCNDV AV2.

## Competing interests

The authors declare that they have no competing interests.

## Authors' contributions

MM and MH conducted all the experiments. SM wrote the manuscript and coordinated the research efforts. YZ conceived the study and edited the paper.
